# Porto Alegre Line predicts lenticulostriate arteries encasement and extent of resection in insular gliomas. A preliminary study

**DOI:** 10.3389/fsurg.2025.1414302

**Published:** 2025-02-10

**Authors:** Gustavo Rassier Isolan, Samir Ale Bark, Jander Moreira Monteiro, Tobias A. Mattei, Kaan Yağmurlu, Rafaela Fernandes Gonçalves, Osvaldo Malafaia, Rafael Roesler, Jurandir Marcondes Ribas Filho

**Affiliations:** ^1^ Graduate Program in Principles of Surgery, Mackenzie Evangelical University, Curitiba, Brazil; ^2^ National Science and Technology Institute for Children's Cancer Biology and Pediatric Oncology—INCT BioOncoPed, Porto Alegre, Brazil; ^3^ The Center for Advanced Neurology and Neurosurgery (CEANNE), Porto Alegre, Brazil; ^4^ Division of Neurological Surgery, St. Louis University, St. Louis, MO, United States; ^5^ Department of Neurosurgery, University of Virginia, Charlottesville, VA, United States; ^6^ Department of Pharmacology, Institute for Basic Health Sciences, Federal University of Rio Grande do Sul, Porto Alegre, Brazil; ^7^ Cancer and Neurobiology Laboratory, Experimental Research Center, Clinical Hospital (CPE-HCPA), Federal University of Rio Grande do Sul, Porto Alegre, Brazil

**Keywords:** insular tumors, lenticulostriate arteries, Optic Chiasm - Insular Recess line, Porto Alegre Line, tumor resection

## Abstract

**Object:**

In insular glioma surgery, lenticulostriate arteries (LSTa) tumoral encasement increases neurological deficits risk despite intensive efforts to preserve the internal capsule's integrity. In this study, we focus on the LSTa relationships with the medial aspect of the insular tumors. We propose a new non-invasive method for LSTa involvement prediction in preoperative MRI (Porto Alegre Line). We compare it with direct intraoperative encased LSTa visualization.

**Methods:**

A retrospective review of our database of 52 patients of insular glioma was performed. In cases with no tumor located medial to Porto Alegre line, our medial resection limit, mainly for the tumor part located next to the limen insula, was the inferior fronto-occipital fasciculus (IFOF), identified through altered speech patterns during electric subcortical stimulation. In cases with no assumed LSTa involvement, the parameter used to stop resection was the confirmation of the corticospinal tract with 10-mA stimulus. The resection limit of tumors placed medially to the Porto Alegre line was intraoperative direct LSTa visualization.

**Results:**

The LSTa involvement was the most critical medial limiting factor in more aggressive tumor resection and an excellent overall survival (*P* = 0.022). In cases in which there were direct intraoperative LSTa encasement visualization, Porto Alegre Line was employed as an MRI preoperative landmark for prediction of LSTa involvement in those patients with Sensitivity, Specificity, Positive Predictive Values of 1, 0.975 and 0.923, respectively.

**Conclusion:**

We have found that LSTa encasement is a limiting factor to reach a satisfactory extent of resection and that Porto Alegre Line can predict it.

## Introduction

Insular gliomas (1–6) are of challenging surgical management because of the complex anatomy of the insula and their proximity to several functional structures such as basal ganglia, internal capsule, speech and language processing cortex and subcortex, as well as the middle cerebral artery and lenticulostriate arteries (LSTa). Gross total resection of these tumors have been shown to impact progression-free survival as well as to contribute to function preservation and quality of life (1–3). Such results can only be obtained through a thorough understanding of the underlying microsurgical anatomy, accurate imaging analysis, language evaluation, and specific brain map techniques (2, 3). Historically, advances in cortical localization were achieved through intraoperative mapping by pioneer neurosurgeons (4, 7–12). Such type of functional mapping, which considers inter-individual variations, has inaugurated the era of surgical brain mapping, which, nowadays, is a *sine qua non* for ensuring safety during surgical resection of such lesions (13). The main advantage of awake brain surgery is the capacity of identifying eloquent brain areas, thereby possibly decreasing postoperative neurological deficits and enabling maximal extent of resection, even with supramaximal resection in some cases (5, 6, 14, 15).

However, brain mapping is quite challenging when the medial aspect of insular tumors does not displace but involves the LSTa. In these cases, even with intensive efforts to preserve the integrity of the internal capsule based on subcortical mapping, there is no assurance that the patient will not experience a new motor or sensory deficit in the postoperative period. This is the main reason why studying the relation of the LSTa with the medial aspect of the insular tumors pre and intraoperatively is of paramount importance. Although the location and deviation of the LSTa on preoperative angiography has already been elegantly shown in a previous study (16), there is still a lack of practical MRI criteria that can predict the involvement of these arteries.

The study aims to describe the current state-of-art for functional mapping during resection of insular gliomas, focusing on the underlying microsurgical anatomy, and the impact of the relationship between the LSTa and the tumor based on the extent of resection, overall survival, and functional outcomes after surgery for insular gliomas. Finally, we describe the “Optic Chiasm - Insular Recess line” (OC-IR line) and Porto Alegre Line on preoperative MRI as an important surrogate marker for prediction of the involvement of the LSTa.

## Materials and methods

A retrospective review of our database of 52 patients of insular glioma operated on by the senior author (GRI) between 2007 and 2018 was performed. Insular gliomas were classified according to Yaşargil (17) and Berger-Sanai classifications (18, 19).

The histological tumor type was defined according to the 2016 World Health Organization (WHO) classification (20). Handedness and language dominance were evaluated employing the Portuguese Edinburgh Handedness Inventory (21, 22). For this study, drug-resistant epilepsy (DRE) was considered a failure of two or more drug therapies in patients with glioma and having at least one epileptic seizure per month. In all cases, we also ran tests at pre and postoperative (one day, three months, and six months) period as follows: Test of Picture Naming (D80 test) and Boston Diagnostic Aphasia Examination (23), and Karnofsky Performance Status (KPS) scale (24).

The Research Ethics Committee of the FEMPAR (FAculdade Evangélica Mackenzie do Paraná) approved this study. Informed consent was obtained from all patients. This study adheres to the principles outlined in the US Code of Federal Regulations, Title 45, Part 46, Protection of Human Subjects, revised January 15, 2009 (25) and the World Medical Association Declaration of Helsinki (26). Microsurgical anatomy was studied and photographed in 5 human cadaveric heads that were prepared in a manner described elsewhere (27, 28).

### Surgical technique

All patients undergoing awake surgery were submitted to a preoperative simulation of the clinical environment's surgical process. During the actual surgery, the patient's head was fixed in a Mayfield frame. Operating microscope and ultrasonic surgical aspirator with a low setting were used in most patients.

Over the years, our technique for resecting the insular tumors has evolved from an approach focused purely on the underlying microsurgical anatomy to a combination of anatomy and functional brain mapping. The first patient of this series, who presented a left insular low-grade glioma (LGG), was operated via transsylvian approach without any type of intraoperative neurophysiological monitoring in 2007 (29).

From 2008 to 2009, we have used a transsylvian approach for all insular tumors with the aid of intraoperative neurophysiological monitoring (including somatosensory and motor evoked potential) (30) with subcortical electric stimulation under general anesthesia to define the medial limit of the resection. We considered a positive electromyographic response in the contralateral body at 10 mA of subcortical stimulation sign of proximity to the motor tract to use this limit parameter as resection limit (31). From 2010 to 2014, for purely insular tumors located in the dominant hemisphere, awake surgery with a transsylvian approach was employed. For insular tumors extended beyond the insula into the frontal and temporal lobes, we used the transcortical approach in those cases in which silent mapping was obtained. During manipulation of the M2 segment of the middle cerebral artery, an intense headache occurred in two patients. Based on this experience, over the years, we have evolved to a transcortical-transopercular approach with subpial resection for all insular tumors in the dominant hemisphere, avoiding direct arterial wall manipulation and manipulation-induced headache (32).

At the beginning of this series, we employed image guidance with navigation in four patients. We have observed that, mainly in larger tumors, the localization parameters were significantly affected after the beginning of the tumor resection due to brain shift. Therefore, in such patients, we currently take the accuracy of navigation with some reluctance. We have not used intraoperative MRI, due to the fact that we identify the resection borders based on function rather than anatomy. On the other hand, we employed intraoperative ultrasound to determine the tumor's position with sulci and gyri. In the past, we used to use intraoperative electrocorticography to evaluate the after-discharge phenomenon during cortical mapping stimulation, but we have recently abandoned it (33). Our parameters of intraoperative cortical and subcortical electrical stimulation have been described elsewhere in the literature (34, 35).

### Identification of the lenticulostriate arteries

We presume that identifying early and pre-operatively the anterior perforated substance via MRI as well as its proximity to the tumor could help ascertain the path of the LSTa across the medial tumor boundaries. The anterior perforated substance can be found in coronal T2-weighted MRI by examining a plane which cuts through the optic chiasm. The optic chiasm in this case is the medial reference, and the insular recess at the anteroinferior aspect of the insular pole is the lateral limit (Optic Chiasm - Insular Recess line - OC- IR line).

**Figure 1:**
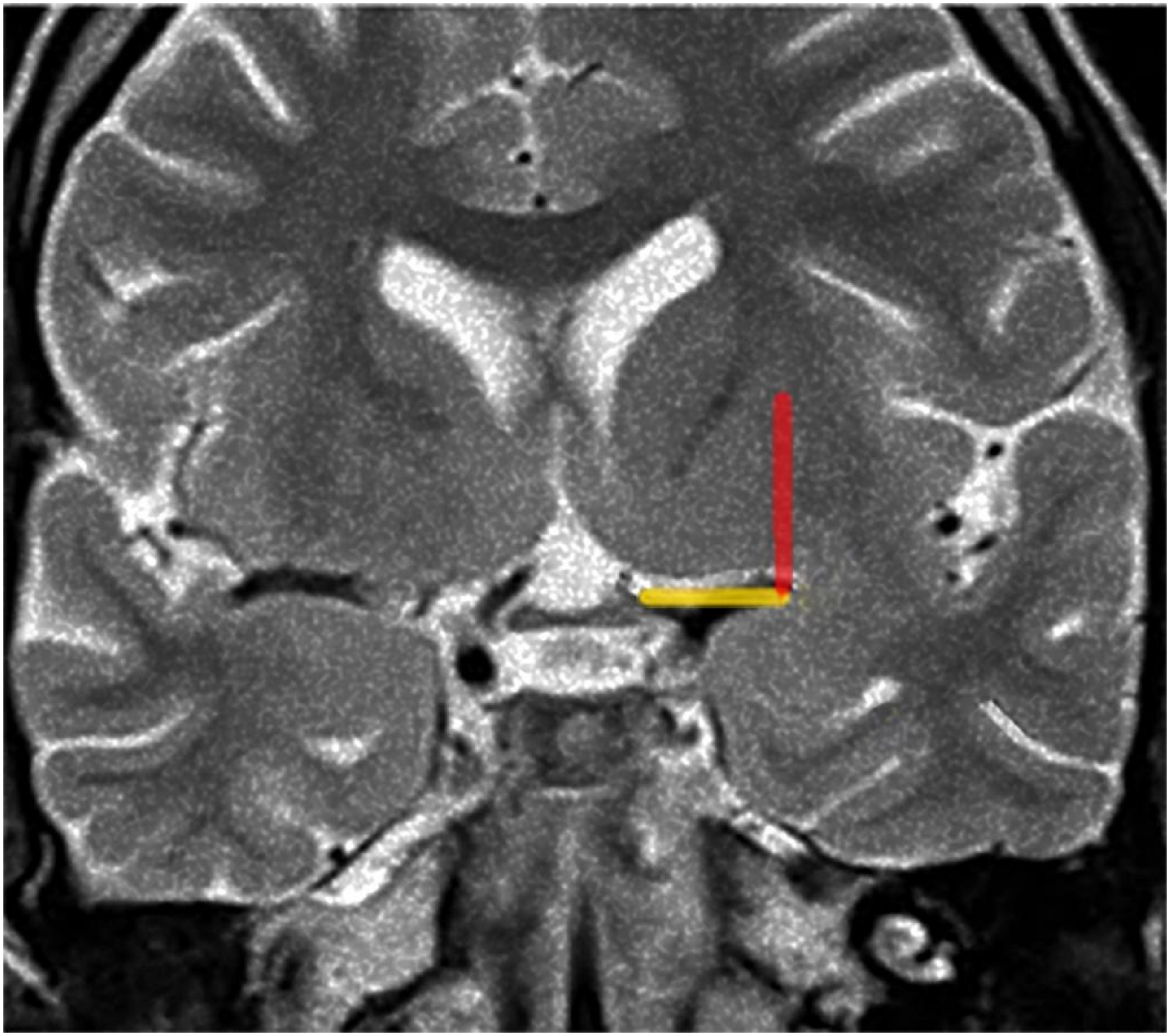
Porto alegre line (red) on coronal T2-weighted MRI reflects indirectaly the lateral limit of the anterior perforated substance, the OC-IR line (yellow). Figure from Isolan et al. (36).

We thus trace a vertical line from the lateral limit of the OC-IR line parallel to the median sagittal plane as the probable lateral LSTa trajectory (Porto Alegre Line) (Figure 1).

Coronal T2-weighted MRI at the level of the optic chiasm as an imaging parameter of the anterior perforated substance. The imaginary line (the Optic Chiasm – Insular Recess line) extends from the optic chiasm medially to the insular recess laterally. It is used to locate the anterior perforated substance (Yellow line). The ascending red line which we call The Porto Alegre Line extends from the lateral end of the OC-IR line and defines the lateral limit of the lateral LSTa within the central core. In cases which the medial border of insular tumor cross this line medially there is a high probability that LSTa will participate in the tumor's evolution.

### Medial limit of tumor resection

**Figure 2:**
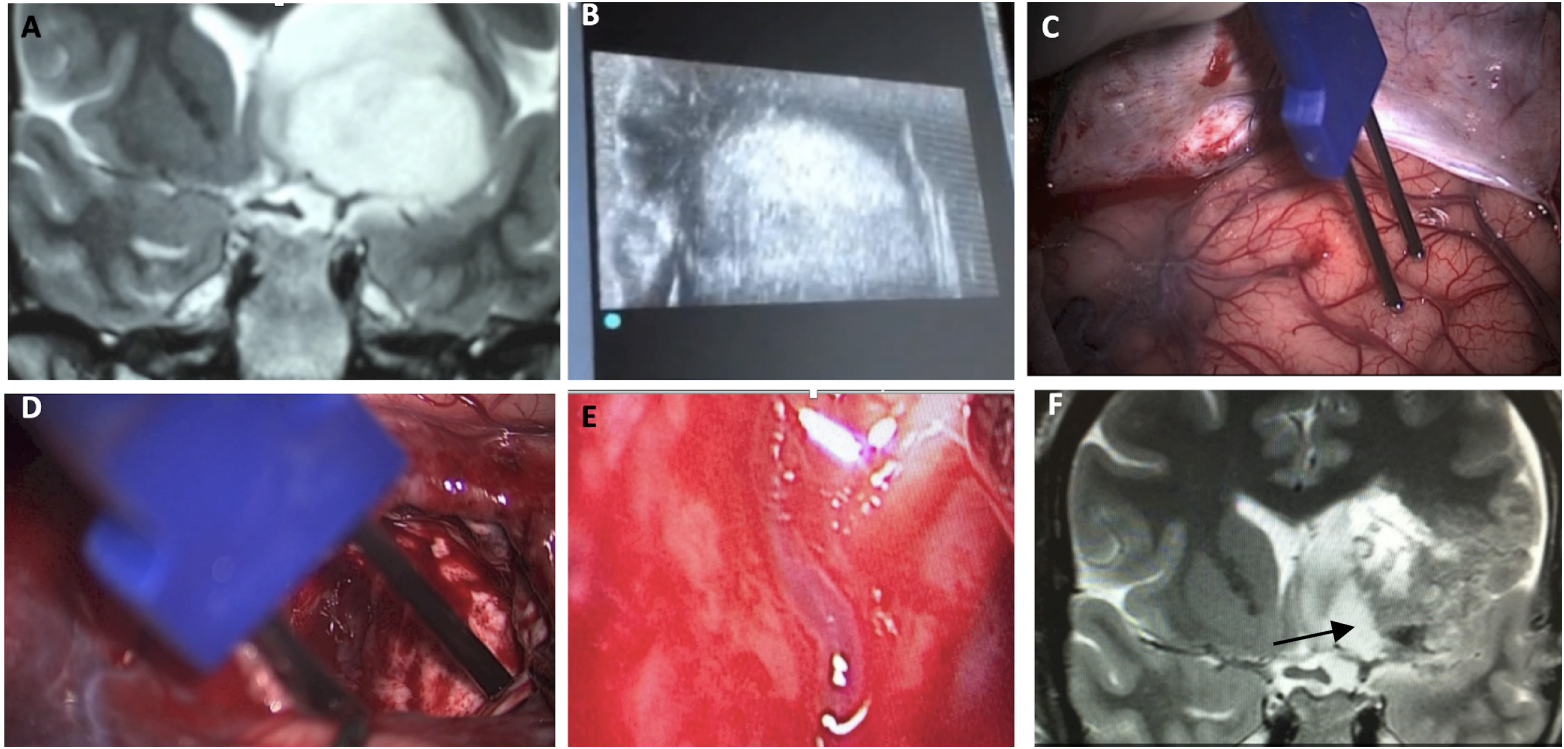
A microsurgical resection of a left insular glioma with LSTa encasement. **(A)** Coronal T2-weighted MRI showing left-sided insular glioma suggesting crossing over the Porto Alegre indicating high probability of LSTa tumoral encasement; **(B)** intraoperative echography; **(C)** cortical brain mapping was silent on the so called “Braca's area”; **(D)** subcortical brain (day 1) showing residual tumor medial to the Porto Alegre line, as it was the medial resection limit. **(E)** Intraoperative view of the LSTa. **(F)** Part of the tumor medial to the Porto Alegre line was not resected (arrow). Figure from Isolan et al. (36).

With insular glioma patients who had undergone awake surgery, our medial limit of resection (the part of the tumor next to the limen insula) was determined mostly by way of altered speech (paraphasia, etc.) under administration of electric subcortical stimulation to the inferior fronto-occipital fasciculus (IFOF). In order to examine the possibility of LSTa being involved at the medial aspect of the tumor, we conducted a careful analysis of coronal and axial T2 and T1-weighted because we understood that the tumors entangling these vessels could not be entirely resected (16, 37–43). In cases in which LSTa involvement was likely, the limiting factor for resection of the medial portion of the tumor was established as the direct intraoperative view of the LSTa. When LSTa within the tumor could be seen, resection was immediately stopped. In patients in which it was assumed that LSTa was not involved, the factor limiting the medial portion resection was the establishment of the corticospinal tract with a 10-mA probe (Figure 2) or paraphasia due to IFOF stimulation in the anterior and inferior part of the tumor.

### Volumetric analysis

Up to 2013, we calculated tumor volume using three largest diameters (D1, D2, and D3) of the tumor taken from T2-weighted MR images along the three principal anatomical planes. Then, we estimated tumor volume using the formula D1 × D2 × D3/2 (44). Since 2014, we have used OsiriX (Pixmeo SARL, Geneva, Switzerland) via stored MR image files in DICOM format (Digital Imaging and Communications in Medicine) (45).

### Statistical analysis

Categorical variables were represented by absolute and relative frequency. Quantitative variables were defined by the median and interquartile range according to the distribution verified by the Shapiro-Wilk normality test.

The proportions of the immediate postoperative deficits and late postoperative deficits (6 months) were compared between the general mapping categories and discriminated by the chi-square test.

For asymmetric variables, we compared the distribution of the extent of resection (EOR) between the categories of LSTa involvement and surgical techniques using the Mann-Whitney and Kruskal-Wallis tests, respectively. Spearman's correlation was performed to verify the degree of relationship between the survival time and the score of the degree of resection (EOR).

Kaplan-Meier analysis was used to describe the mean or median variability and probability at specific survival time points. This description was stratified by symptoms, by Yaşargil's and Berger-Sanai's classifications, and by time ranges from symptoms to diagnosis. Through the Kaplan-Meier analysis, we compared the estimated time distributions using the Log-Rank test. The analysis was performed using the SPSS software (v.25). The classification level used was *p* value < 0.05.

## Results

### Patient demographics

The clinical features of the patient enrolled in this retrospective study are summarized in Table 1. Among 52 patients studied, 30 were male. Most of the tumors were situated on the left side (29 subjects). The most frequent symptom was complex partial seizure.

**Table 1:** Patient's clinical features.

Clinical feature	*N* (%)
Gender	
Male	30 (57.7)
Female	22 (42.3)
Grade	
Grade I	1 (2)
Grade II	30 (57.7)
Grade III	11 (21.1)
Grade IV	10 (19.2)
Side	
Left	29 (55.8)
Right	23 (44.2)
Berger-Sanai's classification	
One zone	7 (13.5)
Two zones	30 (57.7)
Giant	15 (28.8)
Yasargil's classification	
3 (A or B)	28 (53.9)
5 (A or B)	24 (46.1)
Lenticulostriate arteries involvement	
No	39 (75)
Yes	13 (25)
Surgery	
Transcortical	23 (44.2)
Transsylvian	29 (55.7)
Immediate postoperative deficit	
No	33 (63.5)
Yes	19 (36.5)
Late postoperative deficit	
No	45 (86.5)
Yes	7 (13.5)

### Histological findings

A total of 52 patients were enrolled in this study. The histologic analysis was stratified as follows: patients of diffuse astrocytoma (30 subjects) and patients of anaplastic astrocytoma or glioblastoma (22 subjects), 61% and 39% respectively. The mean age for high-grade tumors was 41 (range of 19–68 years), and for low-grade tumors, the mean age was 32 (range of 13–53 years).

### Surgical results and overall survival analysis

According to Berger-Sinai's classification, the tumor was found in one zone in 7 cases (13.5%), two zones in 30 cases (57.7%), and 15 giant tumor cases (28.8%). Considering Yaşargil's classification of insular tumors, 28 patients were classified as type 3 (A or B) and 24 as type 5 (A or B).

When analyzing the survival odds according to the Yaşargil's classification, patients classified as type 3 (A or B) demonstrated a longer survival rate, mean of 121.6 months, and patients of type 5 (A or B) have shown shorter survival rate, mean of 59.1 months (Log-Rank test *p* = 0.008).

According to Berger-Sanai's classification, the survival odds in this series were: when the tumor was found in one zone, mean survival was 98.7 months; whenever the tumor was more invasive and situated in two zones, the mean survival rate was 101.4 months. If the tumor involved more than two zones, the mean survival obtained was 67.9 months.

Regarding extent of resection, we found under the Spearman correlation longer overall survival in the population submitted to more than 90% resection (*R* = −0.037 in low-grade tumors and *R* = −0.169 in high-grade tumors (Table 2).

**Table 2:** The correlation of LSTa involvement with the extent of resection (EOR) and survival and the correlation of EOR via the transsylvian or transcortical and survival rates.

Lenticulostriate arteries involvement			
	No (*N* = 39)	Yes (*N* = 13)	*p* *
EOR	90 [80–100]	70 [50–90]	0.007
Survival (months)	50 [29.5–74.5]	32 [16.5–61]	0.181
Surgery			
	Transcortical (*N* = 22)	Transsylvian (*N* = 29)	*p* **
EOR	85 [70–97.5]	90 [80–100]	0.399
Survival (months)	45 [14–66]	45 [31.25–81]	0.530

* Mann-Whitney test.

** Kruskal-Wallis test.

### Lenticulostriate arteries

**Figure 3:**
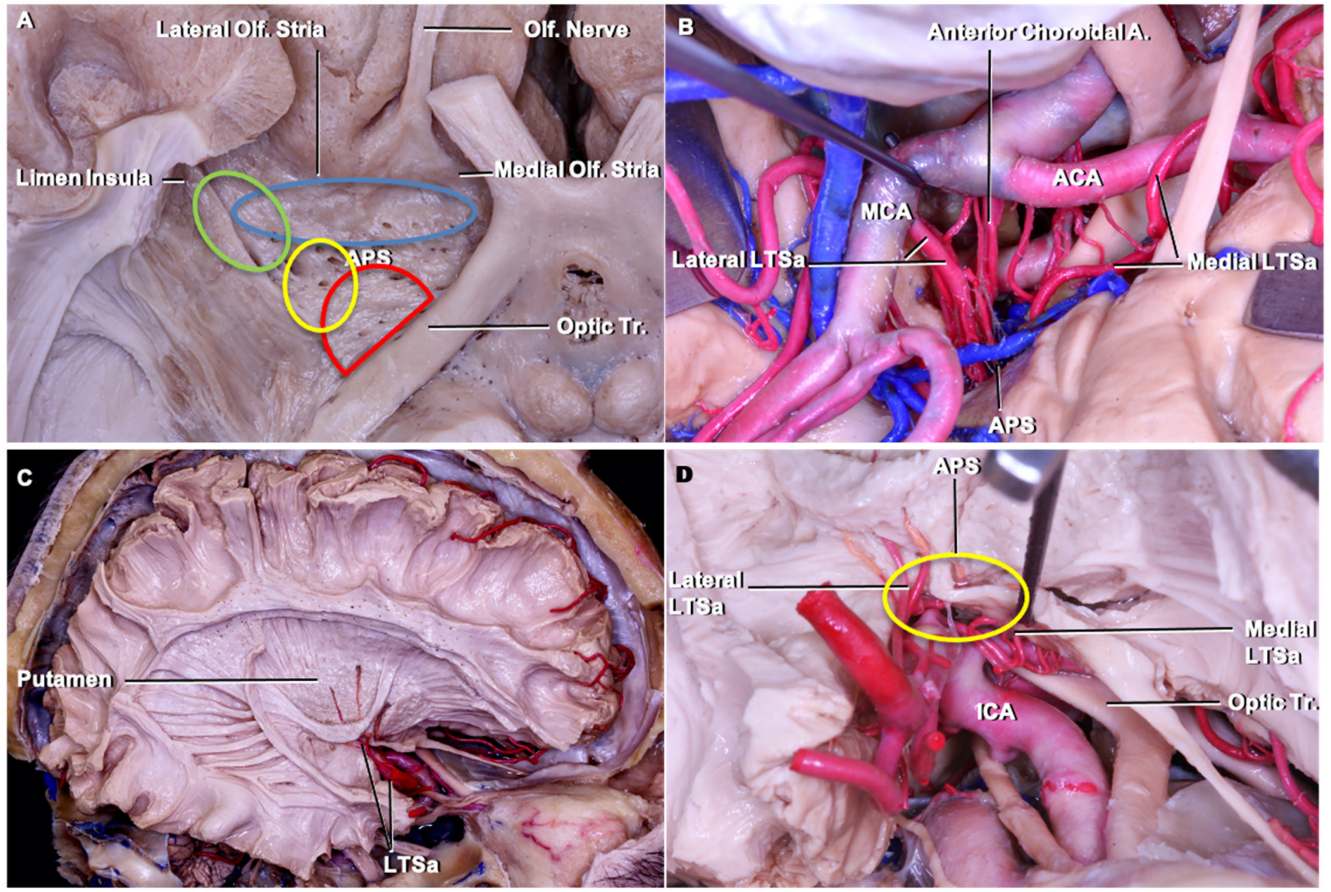
Cadaveric dissection pictures. **(A)** An inferior view of the anterior perforated substance (APS) and LSTa territories. The APS is bordered by medial and lateral olfactory striae, optic tract, and limen (insular) recess. In the APS, the green circle indicates the lateral LSTa, yellow circle indicates the medial LSTa, red circle indicates the branches of the anterior choroidal artery, and blue circle stands for the medial LSTa. The OC-IR is drawn between the optic tract and recess of the limen insula; **(B)** the surgical view of LSTa on the left side. The medial LSTa arises from anterior cerebral artery, and the lateral LSTa arises from the middle cerebral artery; **(C)** white fiber dissection showing the LSTa passing through the APS to supply the basal ganglia; **(D)** enlarged view of C. ACA, anterior cerebral artery; APS, anterior perforated substance; ICA, internal carotid artery; LSTa, lenticulostriate arteries; Tr, tract.

The anterior perforated substance is bordered by medial and lateral olfactory striae, optic tract, and limen recess. The LSTa pass through the anterior perforated substance (APS) to supply the caudate nucleus, thalamus, globus pallidus, putamen, anterior limb, genu and posterior limb of the internal capsule. LSTa subdivided into the medial, intermediate, and lateral groups. The medial LSTa arise mainly from anterior cerebral artery, and both intermediate and lateral LSTa arise mainly from the middle cerebral artery. Microanatomical knowledge of the region and its vascular supply emphasizes the neuroradiological and clinical relation (Figure 3).

**Figure 4:**
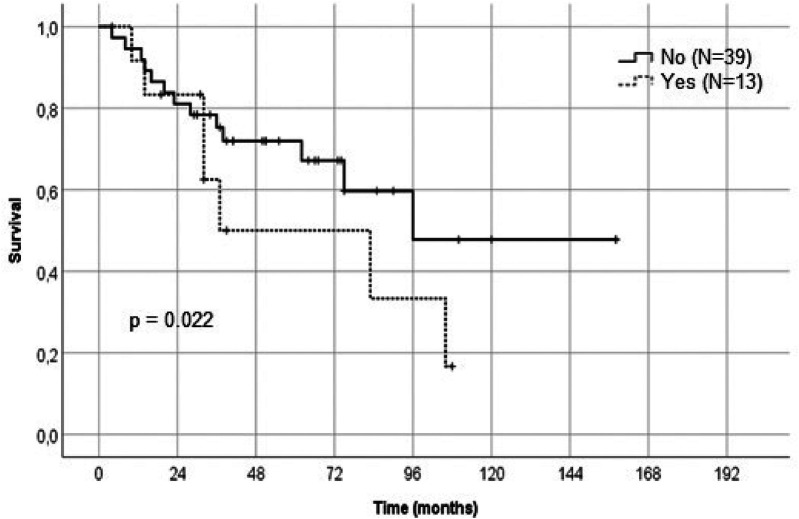
Kaplan-Meier graph showing the relation of the lenticulostriate arteries involvement and survival rate.

**Figure 5:**
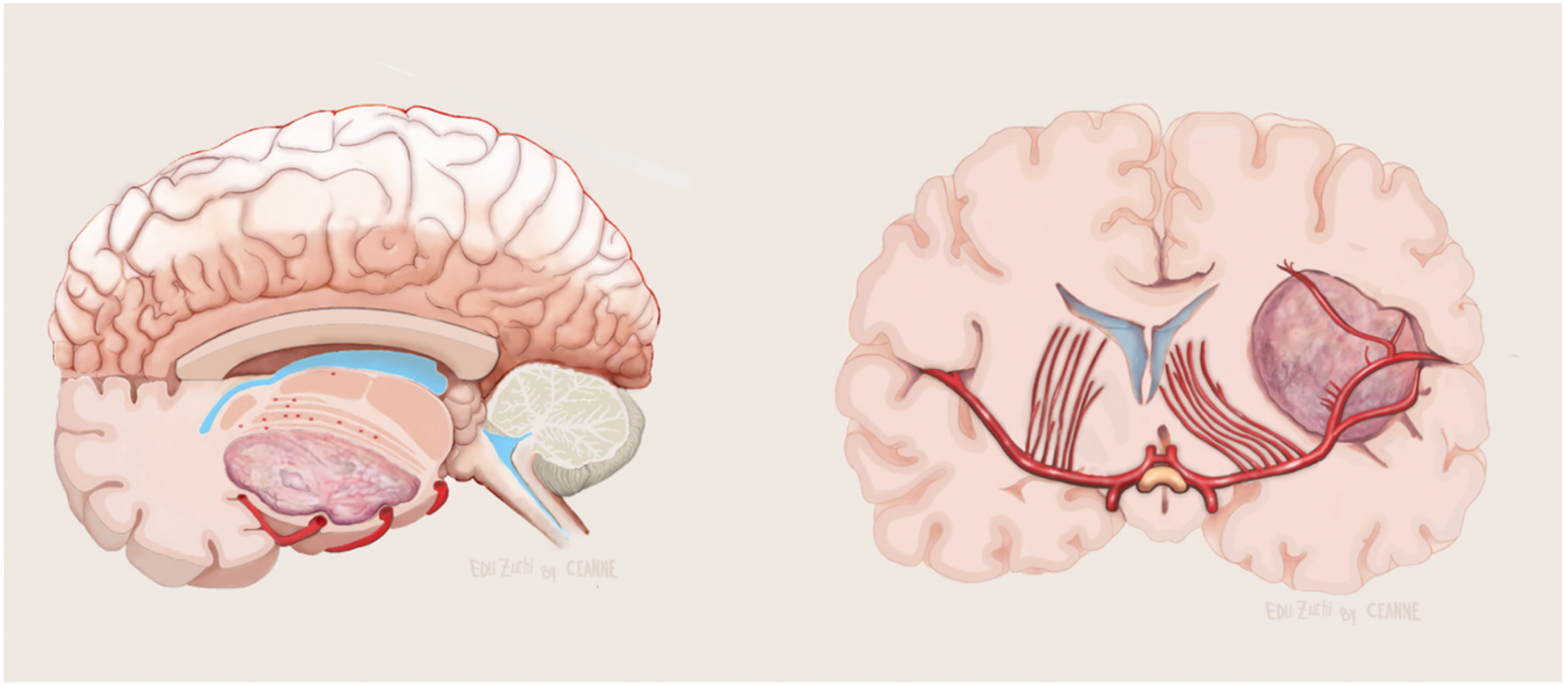
Illustration showing no evolvement of the LSTa by the tumor in axial (left) and coronal (right) view. Figure from Isolan et al. (36).

**Figure 6:**
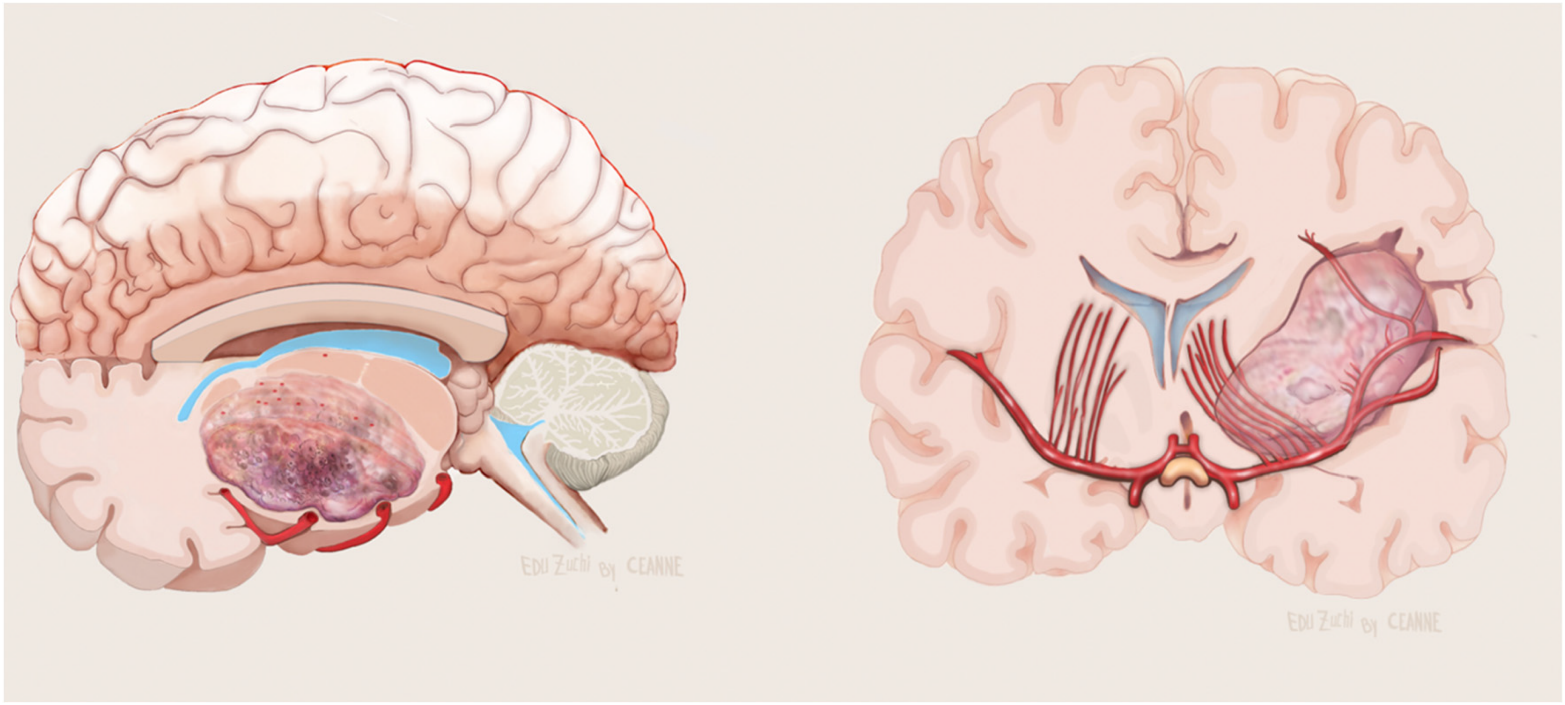
Illustration showing evolvement of the LSTa by the tumor in axial (left) and coronal (right) view. Figure from Isolan et al. (36).

LSTa involvement was determined through preoperative MRI and intraoperative methods, as described above. In 13 patients, LSTa were founded to be involved by the tumor. In 39 patients, there were no involvement of the LSTa, which allowed a more aggressive resection. These findings suggest that tumors with a invasion medially to the anterior perforated substance, that mean's Porto Alegre Line, cannot be submitted to a total resection without causing a severe neurological deficit. Most of these patients have a subtotal resection regardless of the surgical approach (transsylvian or transcortical). The LSTa involvement is probably the most critical medial limiting factor to obtain a more aggressive tumor resection and an excellent overall survival (*p* = 0.022) (Figures 4–6).

We used brain mapping as a parameter to compare immediate and late postoperative neurological deficits. When no mapping was used, 15 patients (40%) developed some immediate neurological deficits, and 6 (16.2%) developed a late deficit. In patients whose motor evoked potentials (MEPs) and somatosensory evoked potentials (SSEPs) were available, 18.2% developed a transient immediate neurological deficit with complete recovery at 3 months. In the awake group, 50% developed immediate motor deficit and none late neurological deficit (total recovery despite the extent of resection) (Table 3).

**Table 3:** Relation of intraoperative brain mapping and neurological deficits.

Brain mapping		
Type	Immediate neurological deficit	Late neurological deficit (3 months)
None	40%	16.2%
MEP and SSEP	18.2%	0
Awake surgery	50%	0

MEP, motor evoked potential; SSPS, somatosensory potential.

Fourteen patients had refractory epilepsy (46). All of them improved their seizures after surgery, being then categorized as Engel I and II.

**Figure 7:**
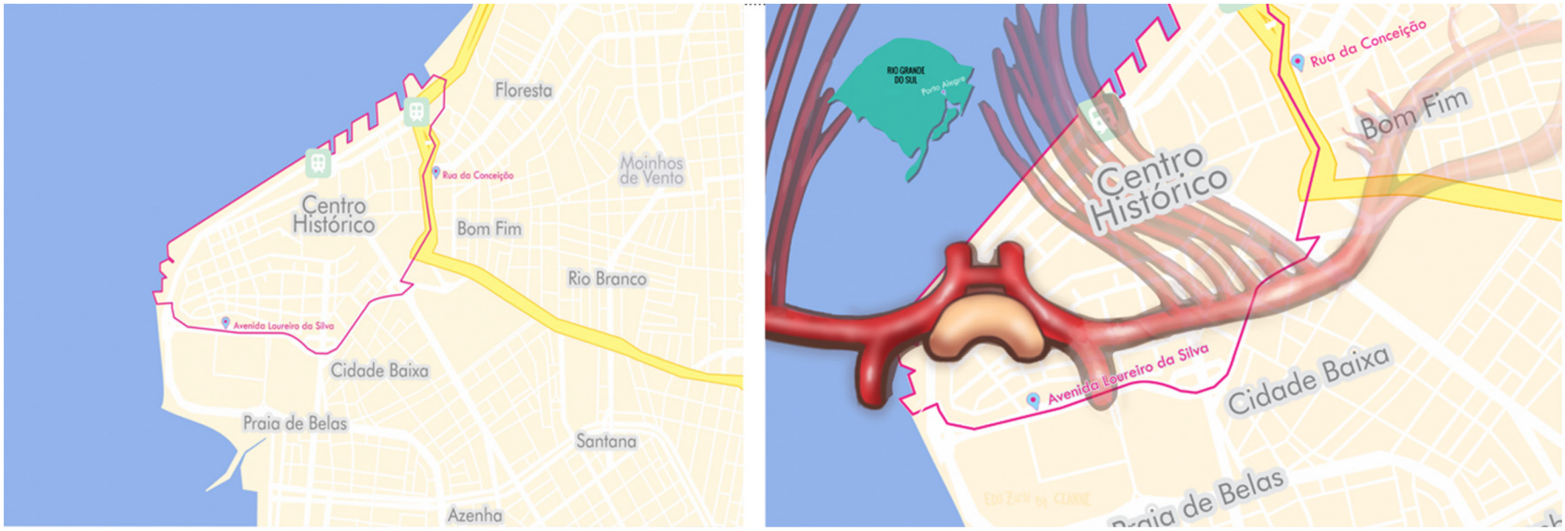
Porto alegrès historic city center (“Centro Histórico”) is one of its busiest neighborhoods and also sees the highest number of muggings. The neighborhood is bounded by Sarmento Leite Street to the east and by the Guaíba River to the west (left). Crossing into the city center runs the risk of falling victim to a crime, much as intruding on insula gliomas can run the risk of injuring the lenticulostriate arteries and causing neurological deficits (right) Figure from Isolan et al. (36).

**Figure 8:**
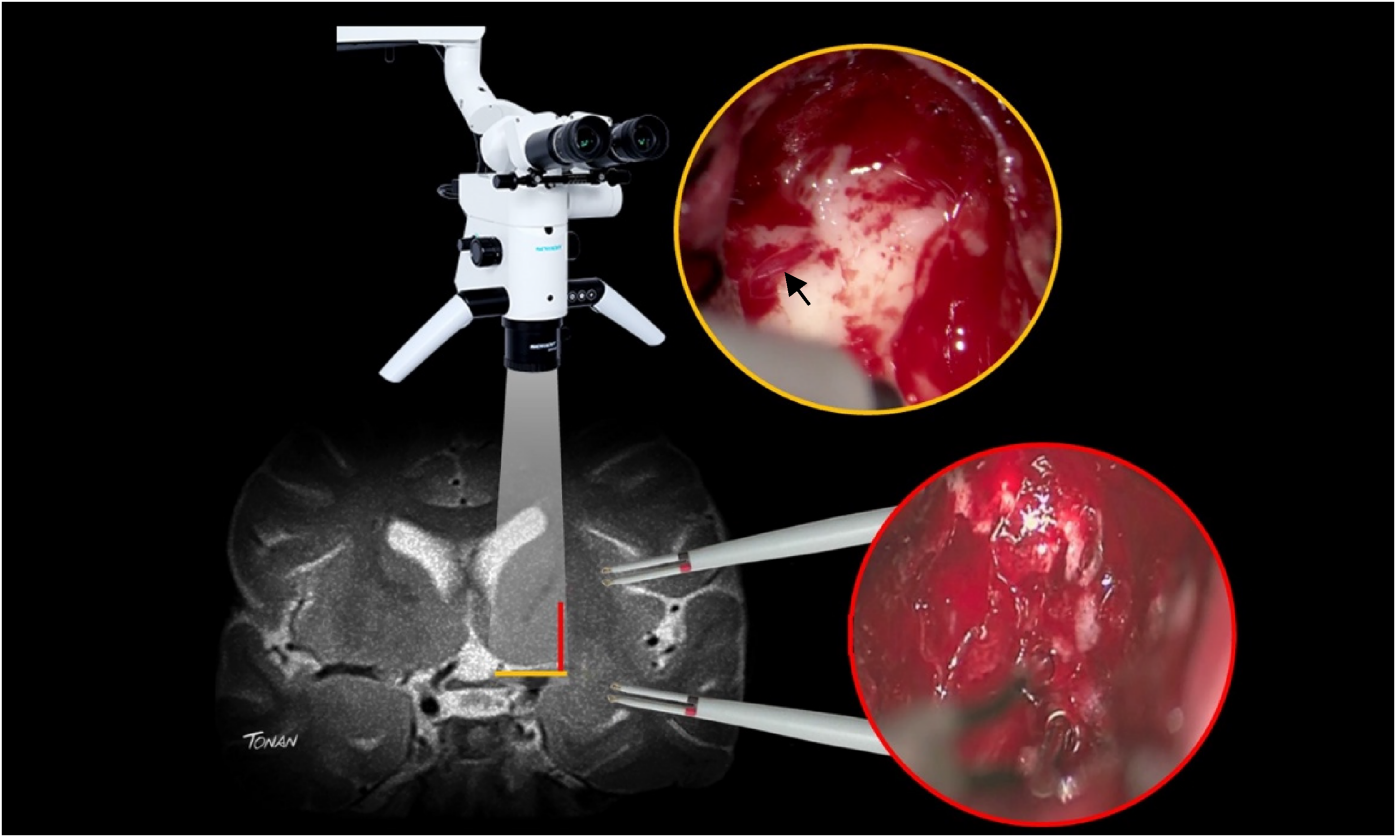
In order to avoid postoperative neurological deficit after insular glioma resection, surgeons have to use subcortical mapping for safe resection of the tumor located lateral to the porto alegre line and microscopic direct visualization of the lenticulostriate arteries (arrow) for safe resection of the tumor located medial to the porto alegre line (red line).

Considering that the LSTa were involved in 13 patients (intraoperative visualization in its superior part with no IFOF distuption), the coronal T2-weighted MRI showed that, using the Porto Alegre Line, as a parameter, the involvement of these arteries could be predicted in all MRIs with tumor located medial to the Porto Alegre Line (Figures 1, 7, 8). In cases in which we did not see the LSTa intraoperatively, the medial limit of resection was based on subcortical mapping and most of these tumors were lateral of the Porto Alegre Line, being a total resection feasable Based on these results, we calculated the Sensitivity, Specificity, Positive Predictive Values of the Porto Alegre Line in predict LSTa encasement by the tumor. Our results were 1, 0.975 and 0.923, respectively.

## Discussion

Microsurgical resection is the gold standard for the treatment of high-grade gliomas. Low-grade gliomas are diffusely infiltrative tumors that generally manifest with seizures in young and professionally active patients. Although surgery of low-grade gliomas has been a controversial subject for decades, it is now also considered the gold-standard therapy (1, 12, 47–52), even in asymptomatic patients (1–3).

Insular gliomas migrate along white matter tracts, progressively invading the surrounding structures. Inevitably, low-grade tumor progresses to high-grade malignancy. A significant delay of malignant transformation of low grades and death can be achieved by appropriate and timely treatment. According to current guidelines, an aggressive protocol with a maximum safe resection, when feasible, is the first line treatment (53–59).

Thus, the management of patients with insular tumors has been dramatically changing during the last two decades, mainly due to technological developments in neuroimaging and the available surgical armamentarium. The benefits provided by these technical advances have generally improved the decision-making process involved in the management of intrinsic brain tumors (60–68).

Awake craniotomy with intraoperative mapping by cortical and axonal stimulation allows minimizing the risk of neurological dysfunction by sparing eloquent surrounding brain structures while improving the resection extent. Cortical and subcortical brain mapping have become a standard of care across multiple subspecialties within Neurosurgery. It is therefore a reliable method in the treatment of insular gliomas.

The technique of intraoperative direct electrical stimulation has been previously described as a safe, precise and reliable method of detection of functional cortical areas (37, 69–72) as well as subcortical supra-tentorial (73), infra-tentorial (74) and spinal pathways (75). Essentially, we began our experience with direct cortical/subcortical electrical stimulation in 2012. From this year on, we were able to obtain a greater extent of resection with simultaneous preservation of functional areas at the same time. We have found, analyzing our cases submitted to the awaked protocol, that all the patients operated with gross total resection philosophy were functionally preserved at six months postoperatively.

When targeting insular tumors, some neurosurgeons advocate the traditional transsylvian approach. After the opercular split and exposing the middle cerebral artery, frontal and temporal branches, superior and inferior circular sulci, limen insulae, and anterior perforated substance, the tumor was resected in-between MCA branches. Other neurosurgeons advocate a transcortical approach. In a transcortical approach, after cortical mapping, one or more delicate and limited subpial dissection is carried out until the superior or inferior circular insular sulcus, exposing the superior or inferior insular surface. In our series, twenty-nine patients were operated on with the transsylvian route and twenty-two patients with the transcortical route. We could not find a statistical significance to establish a clear correlation between surgical approaches and long-term survival or extent of resection. In theory, transcortical approach is a tumor-directed approach but has the disadvantage of a poor arterial control, especially the lateral lenticulostriate group. In our surgeries, we noted that this was not a problem because we could predict the LSTa encasement using the Porto Alegre Line. In the transsylvian approach, most of the vessels are directly seen but, just because of that, vascular injury may be a possible associated complication. Another disadvantage of this approach is that sometimes the posterior third of sylvian fissure cannot be appropriately split while preserving the superficial sylvian vein and its anastomosis with Labbé or Trolard veins. This is especially true in a tumor located in zone 2 of Berger-Sanai's classification.

Especially in awaked surgery, vascular manipulation in transsylvian approach has produced severe headache in two of our patients, as mentioned before. This was one of the reasons we switched to the transcortical approach in awake patients. It is significant to note that surgery in the insula may induce postoperative hemiparesis and speech impairment after dominant insula infarction or aggressive resection, and mutism and apraxia after non-dominant insula damage (76, 77).

### Insular glioma classification

There are two main classifications for insular gliomas. Yaşargil's first extended series of limbic and paralimbic tumors included 57 insular and parainsular tumors (17). After the advent of microsurgical techniques, these first encouraging results convinced many neurosurgeons to take a renewed interest in insular tumor surgery. Yaşargil et al. proposed a classification system based on whether a tumor was restricted to the insula (type 3), was part of the insula (type 3A), or was included in the adjacent operculum (type 3B). Tumors Type 5A and 5B were those involving one or both of the paralimbic orbitofrontal and temporopolar areas, respectively.

Berger and Sanai have developed a quadrangular system to classify tumors in zone I to IV according to their major locations (51). The system considered a plane traced over the sylvian fissure where the insula straddle intersected by a perpendicular plane through a projection of the foramen of Monro. This classification is essential because the tumor in zone 2 (superior and posterior part of the insula) is better resect with transcortical approach in order to avoid brain retraction and possible venous infarction of posterior third of the superficial sylvian veins and its anastomosis.

In our series, we observed that type 3 tumors in Yaşargil classification could be resected with a transinsular approach. Notable, despite the decision making of surgical approaches (transcortical or transsylvian), survival rate was affected by the LSTa encasement.

### Arterial relationships

A few key factors significantly impact the extent of resection at the medial boundary of insular tumors. The insular cortex is oriented laterally and acts as the medial wall of the operculoinsular compartment. The gray matter that lies medially to the insular cortex contains claustrum and the putamen; the internal capsule also lies medially, and is quite visible in MRIs of healthy subjects. When a tumor is present, the claustrum is difficult to identify because of its narrow form and flimsy structure. The putamen is more rigid than the insular cortex and can usually resist glioma's spread (38). Even though some insular tumors have obvious limits when viewed in MRIs, others appear to intrude on the putamen and at times the internal capsule. The MCA's branches (M2) can normally be found laterally to the insular surface. Tiny M2 branches penetrate the lateral surface in order to serve the insular cortex, the claustrum, and the external capsule. The lenticulostriate arteries (lateral group) are branches of the M1 (sphenoidal) segment and they cross superiorly, penetrating the anterior perforated substance to supply blood to the substantia innominata, putamen, globus pallidus, head and body of the caudate nucleus, internal capsule as well as the adjacent corona radiata, and the lateral portion of the anterior commissure (44, 78, 79). These number 1–15 per hemisphere, with an average of 7.75. The diameter of LSTa ranges from 0.1 to 1.5 mm (78, 80, 81). There is normally no anastomosis between any LSTa. The lateral view of LSTa appears fan-shaped and covers the lateral face of the internal capsule.

The sylvian fissure has to be opened, otherwise a trans-opercular access has to be performed, to reach the insular glioma. While tumor removal advances medially, the LSTa can become compromised and lead to ischemia of the basal ganglia and internal capsule (45). For this reason, preserving the LSTa is of primary importance. Some tumors may grow and infiltrate medial structures including the LSTa. If this occurs, stopping resection is recommended and the LSTa can be regarded as the primary limiting factor. In the part of the insula near to insular recess (inferior and anterior) identification of the IFOF provides the medial limit of the resection. On the other hand, on the superior and posterior parts of the tumor in order to perform an uneventful insular tumor resection, the vertical plane of the intraparenchymal course of the LSTa should define the medial limit of resection (30, 44, 82, 83).

In Moshel series, the location of the displaced LSTa on preoperative angiograms was employed to locate tumors extending medially into the putamen and globus pallidus, and thus amplifying the risk of postoperative deficit. This was found to be highly correlated when the medial tumor boundaries were established through MRIs superimposed on stereotactic cerebral arteriograms. In a series of 38 insular glioma patients, gross total resection was achieved more often when tumors were lateral to the LSAs, when they medially shifted the LSAs, and when they had well-defined tumor borders on T2-weighted MRIs. In cases in which tumors extended medially to the LSTa, EOR was subtotal (16).

Identifying the LSTa intraoperatively within the sylvian vallecula does not necessarily indicate their intraparenchymal route as the tumor can medially displace arteries here. Yaşargil found that low-grade gliomas initially extended within the confines of the anatomical limbic system's, which suggests that resection will be complete when LSTa are observed intra-operatively or when the common white matter is found covering the putamen (84). The problem of this technique is to disrupt the IFOF before see the LSTa, which can happen in the inferior and anterior part of the tumor and cause language deficit, mainly in the dominant hemisphere.

These are our images and anatomical brief for LSTa during resection of insular gliomas. Detailed knowledge of LSTa's origin, path, and spread is essential to successful resection, particularly the lateral group. Their identification or, at least, anatomical knowledge of their path has a direct effect on the limits of tumor resection and on preservation of function. Starting from the dorsal aspect of the M1 segment of the middle cerebral artery, the LSTa enter the lateral two-thirds of the anterior perforated substance of the basal forebrain, a quadrilateral area of grey matter located anterolateral to the optic tract and posterior to the gyrus rectus and olfactory trigone. It is medially limited by the optic chiasm and laterally by the lateral olfactory striae. After going across the anterior perforated substance, the LSTa extend into the lateral thirds of the anterior commissure, the lateral portion of the globus pallidus, the superolateral two-thirds of the head and whole body of the caudate nucleus, and most of the putamen, then outward towards and including the external capsule, the superior portion of the whole anterior limb and superior portion of the posterior limb of the internal capsule and periventricular white matter (corona radiata) at the angle of the lateral ventricle. Isolated tumor cells in insular gliomas can penetrate intact parenchyma and generate tumor tissue encasing the LSTa.

Certain clinicians advocate stereoscopic angiography and computerized tumor reconstruction, MR angiography, and CT angiography to estimate the position of the LSTa (16). In spite of the soundness of these studies, we have demonstrated that coronal T2-weighted MRIs Porto Alegre Line can predict the LSTa's location and route to great utility. This method was able to anatomically locate LSTa medially encased by tumors in 13 subjects, or 25% of cases. In 75%, the medial tumor border was confined to the normal white matter lateral to the putamen. Over 90% the tumors were resected from the latter patients. The presence of LSTa was a limiting factor to a reasonable extent of resection and we recommend that tumors encasing these arteries must be left intact due to the high risk of permanent postoperative functional deficit.

Another possible etiology of internal capsule and corona radiata infarction could be, at least theoretically, lesion or coagulation of the long perforating arteries (LPra) (85). The LPra originate in the M2 segment of the MCA vascularizing the insular cortex. A majority of these are short, however, the LPra, most commonly located in the posterior half of the central insular sulcus and the long gyri, can reach the corona radiata and/or more deeply in 36% of cases. Some pass through the fibers of the corona radiata and reach the lateral ventricular body's ependyma. In our experience, we coagulated long perforating arteries (LPra) in the posterior part of the insula without any postoperative stroke observed.

The external capsule is the limit of the regions supplied by the short insular arteries. No anastomosis occurs among the insular and lenticulostriate arteries. Although the LPra could be the reason for a postoperative motor deficit after insular glioma resectoin involving LSTa preservation,. Again, we did not find this to be the case with any of our patients, even after coagulating all insular perforator arteries using the transsylvian approach.

### The city of Porto Alegre and the meaning of the Porto Alegre Line

Porto Alegre anchors Brazil`s fifth-largest metropolitan region of 4.4 million inhabitants and is the capital of its southernmost state, Rio Grande do Sul. This multicultural city sits at the junction of five rivers and has become a significant port in one of the country`s chief industrial and commercial regions. It contains several public and private universities as well as renowned public and private hospitals. In the 2010s, Porto Alegre suffered a growing wave of violence and in 2017 was ranked the world`s 39th most violent city (86). Fortunately, the incidence of violent crime has been dropping steadily since then (87, 88).

Porto Alegrès Historic City Center is one of its busiest neighborhoods and also sees the highest number of muggings. The neighborhood is bounded by Sarmento Leite Street to the east and by the Guaíba River to the west (36). Crossing into the city center runs the risk of falling victim to a crime, much as intruding on insula gliomas can run the risk of injuring the lenticulostriate arteries and causing neurological deficits.

Our study has some intrinsic limitations, such as the small number of cases with awake surgery. To the small number of patients, as expected in dealing with rare diseases, we have not performed multivariate analysis. In the near future we consider exploring the integration of the Porto Alegre Line with other advanced imaging modalities or machine learning algorithms to enhance predictive accuracy.

## Conclusion

Adding cortical and subcortical mapping to microsurgical anatomy x-ray view knowledge is paramount to face insular glioma surgery in the modern era and to not put the patient on jeopardy (36, 89–91). We have found that LSTa encasement is a limiting factor which impacts the EOR of insular gliomas. Thus, future attempts to classify such lesions should consider the encasement of the LSTa among its prognostic factors for overall survival and EOR of these tumors, possibly enabling the surgeon to anticipate a subtotal resection. The preliminary data obtained in this study is expected to provide a foundation for future studies on the significance of the LSTa in insular glioma surgery.

## Data Availability

The raw data supporting the conclusions of this article will be made available by the authors, without undue reservation.
